# Temperature Increase Negatively Affects the Fatty Acid Bioconversion Capacity of Rainbow Trout (*Oncorhynchus mykiss*) Fed a Linseed Oil-Based Diet

**DOI:** 10.1371/journal.pone.0164478

**Published:** 2016-10-13

**Authors:** Julie Mellery, Florian Geay, Douglas R. Tocher, Patrick Kestemont, Cathy Debier, Xavier Rollin, Yvan Larondelle

**Affiliations:** 1 Institut des Sciences de la Vie, Université catholique de Louvain, Louvain-la-Neuve, Belgium; 2 Unité de Recherche en Biologie Environnementale et Evolutive, Université de Namur, Namur, Belgium; 3 Institute of Aquaculture, School of Natural Sciences, University of Stirling, Stirling, Scotland, United Kingdom; 4 Earth and Life Institute, Université catholique de Louvain, Louvain-la-Neuve, Belgium; Universitat Politècnica de València, SPAIN

## Abstract

Aquaculture is meant to provide fish rich in omega-3 long chain polyunsaturated fatty acids (n-3 LC-PUFA). This objective must be reached despite (1) the necessity to replace the finite and limited fish oil in feed production and (2) the increased temperature of the supply water induced by the global warming. The objective of the present paper was to determine to what extent increased water temperature influences the fatty acid bioconversion capacity of rainbow trout (*Oncorhynchus mykiss*) fed a plant-derived diet. Fish were fed two diets formulated with fish oil (FO) or linseed oil (LO) as only added lipid source at the optimal water temperature of 15°C or at the increased water temperature of 19°C for 60 days. We observed that a temperature increase close to the upper limit of the species temperature tolerance range negatively affected the feed efficiency of rainbow trout fed LO despite a higher feed intake. The negative impact of increased water temperature on fatty acid bioconversion capacity appeared also to be quite clear considering the reduced expression of *fatty acid desaturase 2* in liver and intestine and the reduced Δ6 desaturase enzymatic activity in intestinal microsomes. The present results also highlighted a negative impact of increased temperature on the apparent *in vivo* enzymatic activity of Δ5 and Δ6 desaturases of fish fed LO. Interestingly, this last parameter appeared less affected than those mentioned above. This study highlights that the increased temperature that rainbow trout may face due to global warming could reduce their fatty acid bioconversion capacity. The unavoidable replacement of finite fish oil by more sustainable, readily available and economically viable alternative lipid sources in aquaculture feeds should take this undeniable environmental issue on aquaculture productivity into account.

## Introduction

According to climate models, climate change will be associated with a gradual rise of surface temperature from 1 to 4°C by 2100 [1]. An increase of 0.2 to 2°C in water temperature has already been reported in lakes and rivers in Europe, North America and Asia [2]. This will affect freshwater fish communities and fisheries [3] and could impact directly and indirectly on aquaculture productivity [3, 4]. Increased water temperature is known to directly affect several physiological processes in fish, including growth [5, 6], basal metabolic rate [6, 7], digestive physiology [8, 9], swimming performance [10], cardiac function [11], reproductive performance [12], and oxidative stress management [13]. The detrimental effects of increased temperature on fish above optimum temperature for growth may be explained notably via its influence on biochemical reaction rates and via reduced oxygen availability and transport with increased temperature, which can therefore not respond to the higher tissue demand in oxygen [3, 14]. Moreover, tolerance to water temperature variation depends highly on fish species, previous life stage conditions and current physical resources [3, 10, 15]. Aquaculture productivity could also be affected indirectly by climate change through changes in precipitation patterns, river flow, drought frequency, increased pollutant toxicity and disease occurrences [1, 2, 4, 16]. In addition, aquaculture may suffer from reduced dietary ingredient availability, both in terms of fish meal and fish oil, resulting from a climate-associated impaired ocean productivity [2], and in terms of terrestrial ingredient alternatives, resulting from impaired crop production [1].

Fish oil is one of the most valuable ingredients used in fish feed production [4, 17, 18], owing to its lipid profile perfectly matching with salmonid fatty acid requirements [19, 20]. In addition, the use of fish oil in aquaculture enables farmed fish rich in omega-3 long chain polyunsaturated fatty acids (n-3 LC-PUFA), namely eicosapentaenoic acid (EPA, 20:5n-3) and docosahexaenoic acid (DHA, 22:6n-3), to be produced for the human consumer. The n-3 LC-PUFA are known to be involved in fundamental physiological processes and to have many positive effects on human health [21, 22]. As fish and seafood are the richest sources of n-3 LC-PUFA [21, 22] and these fatty acids are considered as semi-essential or essential for humans, the continuous supply of high nutritional value fish is of utmost importance. However, fish oil has become rare and expensive, economically and environmentally speaking, as it is a finite and limited marine resource [17, 19]. This ingredient is thus progressively replaced by more sustainable alternative lipid sources in fish feed. In this context, plant-derived oils are considered as promising alternatives [18] and have already been included in commercial feeds without compromising fish growth performance [23–26]. Nevertheless, plant-derived oils do not contain n-3 LC-PUFA, which decreases the amount of EPA and DHA in the feeds and, consequently, the farmed fish, compromising their nutritional benefits to human consumers [18, 23–26]. Interestingly, although all plant-derived oils are devoid of n-3 LC-PUFA, a few such as linseed oil contain a high percentage of the n-3 LC-PUFA precursor, namely alpha-linolenic acid (ALA, 18:3n-3) [18, 19].

**Fig 1:**
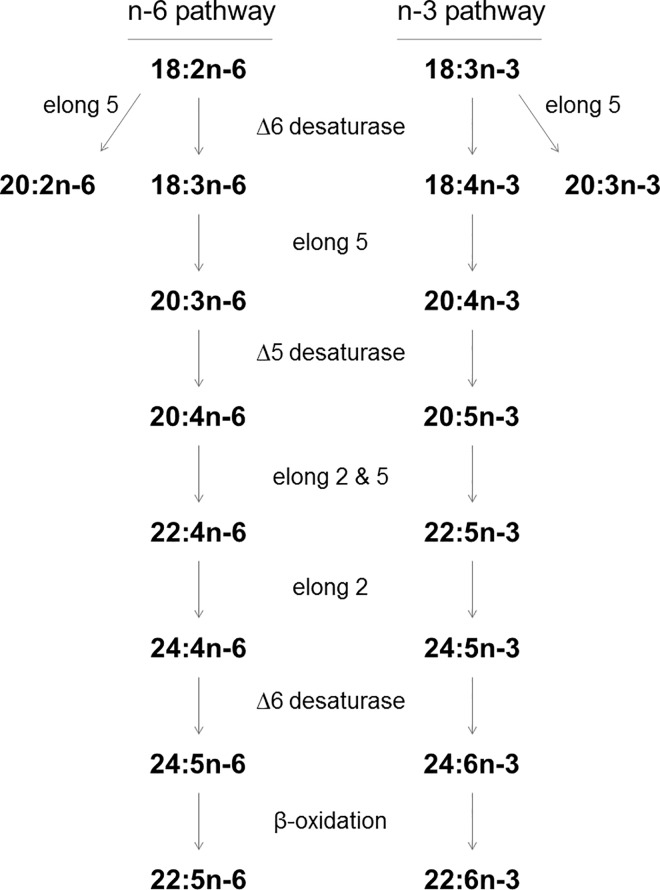
Pathways for endogenous elongation and desaturation of 18:2n-6 and 18:3n-3 to produce n-6 and n-3 long chain polyunsaturated fatty acids, respectively. The enzymatic system acts on both pathways in parallel requiring Δ6 and Δ5 desaturases and fatty acyl elongases 2 and 5. Elong, fatty acyl elongase.

Since salmonid farming consumes about 60% of the total fish oil used in commercial aquafeeds [27], research on fish oil replacement by plant-derived oils is particularly focused on these species. Among the salmonids, rainbow trout (*Oncorhynchus mykiss*) is an important cultured fish species in temperate regions [4, 17, 28]. Moreover, rainbow trout possesses a good capacity to endogenously produce EPA and DHA from ALA, via the n-3 fatty acyl desaturation and elongation pathway requiring Δ6 and Δ5 desaturases, and fatty acyl elongases (Fig 1), mainly in liver, intestine and brain [19, 29–31]. The endogenous production of arachidonic acid (ARA, 20:4n-6) from linoleic acid (LA, 18:2n-6) through the omega-6 (n-6) pathway takes place in parallel with the n-3 pathway utilising the same enzymatic system (Fig 1). The n-3 fatty acid bioconversion capacity of rainbow trout could therefore be exploited in order to continue providing the human consumer with fish rich in EPA and DHA, while replacing fish oil by linseed oil, rich in ALA, in feed. Promising results have already been reported [23–26], although the n-3 LC-PUFA content reported in these studies was lower than the n-3 LC-PUFA content of fish fed with fish oil. Interestingly, Tocher *et al*. [32] showed a significant LC-PUFA synthetic capacity in isolated hepatocytes of Atlantic salmon (*Salmo salar*) fed a plant-derived oil diet during its early growth stages, and the progressive decrease of that capacity until a size of 2 kg. Even at the early growth stage, the efficiency of bioconversion is of great interest from a nutritional point of view since it is during these days that the fish synthesise a significant part of their n-3 LC-PUFA from the corresponding precursors, in the case of permanent feeding with plant-derived oils, a large part of the newly synthesised n-3 LC-PUFA being expected to be kept in the fish body until a marketable size.

Little is known about the impact of elevated water temperature on the fatty acid bioconversion capacity of rainbow trout. Rainbow trout possesses a very narrow optimal temperature growth range (15–16°C) but tolerates temperatures between 4°C and 20°C [28]. Increased water temperature in aquaculture will undoubtedly affect rainbow trout physiology and metabolism [4], and may thus modify its lipid bioconversion capacity, impacting the n-3 LC-PUFA content of fish fed plant-derived oils. Several studies have reported the combined influence of dietary lipid source and temperature in salmonids [33–37] and other fish species [5, 7, 38, 39]. Recent studies evaluating the impact of different ARA/EPA ratios and water temperature (10°C or 20°C) on Atlantic salmon growth and lipid metabolism concluded that increased temperature induced increased feed intake, hepatic ARA accumulation and apparent *in vivo* fatty acid β-oxidation. Conversely, decreased apparent *in vivo* activity of Δ6 desaturase and decreased expression of genes involved in fatty acyl desaturation (Δ5 and Δ6 desaturases) were observed [33, 34]. In contrast, Tocher *et al*. [35] observed reduced fatty acid β-oxidation, and lower elongation and desaturation enzymatic activities in isolated hepatocytes and enterocytes from rainbow trout held at a water temperature of 15°C in comparison with 7 and 11°C, and fed a palm oil-based diet.

Considering the above, the objective of the present paper was to determine to what extent increased water temperature could influence the fatty acid bioconversion capacity of rainbow trout fed a plant-based diet by analysing fish growth, whole fish fatty acid composition, apparent *in vivo* enzymatic activities of desaturases and elongases determined by the whole body fatty acid balance method [40, 41], hepatic and intestinal gene expression and intestinal Δ6-desaturase enzymatic activity.

## Materials and Methods

### Ethics statement

The experimental design was approved by the Animal Care and Use Committee of the Université catholique de Louvain (Permit number: 123203) applying the EU legal frameworks relating to the protection of animals used for scientific purposes (Directive 86/609/CEE) and guidelines of Belgian legislation governing the ethical treatment of animals (Decree M.B. 05.01.1994, November 14^th^, 1993). The feeding and digestibility trials were set up at the “Plateforme technologique en biologie aquicole Marcel Huet” (Université catholique de Louvain, Louvain-la-Neuve, Belgium), which is certified for animal services under permit number LA 1220034. All fish manipulations were performed under anaesthesia with 2-phenoxyethanol (Sigma-Aldrich, St Louis, MO, USA, 0.3 ml/l) and, if necessary, fish were killed with excess 2-phenoxyethanol. All efforts were made to minimise fish number and suffering. No clinical symptoms were observed within or outside the experimental periods.

### Experimental diets

Two iso-energetic, iso-nitrogenous and iso-lipidic experimental diets were formulated to meet the nutrient requirements of rainbow trout [20]. Diets differed according to the added lipid source: the control diet (FO) was formulated with cod liver oil whereas the plant-derived diet (LO) was based on linseed oil. The detailed formulations and the proximate and fatty acid compositions are shown in Tables 1 and 2, respectively. The FO diet was particularly rich in n-3 LC-PUFA in which EPA (6.6 mg/g of dry matter (DM)) and DHA (10 mg/g DM) were the major fatty acids. In contrast, the LO diet contained mainly ALA (38.6 mg/g DM) and residual levels of n-3 LC-PUFA (1.2 mg/g DM). Chromic oxide (Sigma-Aldrich) was added at 10 g/kg DM to each experimental diets intended for the digestibility trial in order to serve as indigestible marker. The experimental diets were produced as previously described [42]. Briefly, the dry components were homogenised (SM 20, Guangzhou Both-Win, Guangdong, China) before and after the oil addition and then after the water addition. After cold extrusion (HI 2251, Simplex, Paris, France) and freeze-drying, the diets were stored at -20°C until feeding or analysis.

**Table 1:** Components (g/kg of dry matter) and chemical composition of the control diet (FO) and the linseed oil diet (LO).

	FO	LO
*Components *		
Cod muscle meal ^1^	100.3	100.3
Blood meal ^2^	130	130
Wheat gluten meal ^3^	250	250
Gelatin ^4^	50	50
Lysine.HCl ^4^	10	10
Modified starch ^5^	209.7	209.7
Glucose ^4^	25	25
Agar ^4^	10	10
Carboxymethylcellulose ^4^	40	40
Cellulose ^4^	10	10
Cod liver oil ^6^	90	0
Linseed oil ^7^	0	90
Vitamin premix ^8^	10	10
Mineral premix ^9^	65	65
*Chemical composition *		
Crude ash (% of DM)	7.3	7.4
Crude protein (% of DM)	49.4	49.9
Crude lipid (% of DM)	10.8	11.5
Gross energy (MJ/kg DM)	21.5	21.6

DM, dry matter.

^1^ Snick Euroingredients, Beernem, Belgium.

^2^ Veos, Zwevezele, Belgium.

^3^ Dumoulin, Seilles, Belgium.

^4^ Sigma-Aldrich, St-Louis, MO, USA.

^5^ Roquette, Lestrem, France.

^6^ Certa, Waregem, Belgium.

^7^ Eden Reform, Heimertingen, Germany.

^8^ The vitamin complex (g/kg premix): retinol acetate 0.67, ascorbic acid 120, cholecalciferol 0.1, α-tocopherol acetate 34.2, menadione 2.2, thiamin 5.6, riboflavin 12, pyridoxine 4.5, calcium pantothenate 14.1, *p*-aminobenzoic acid 40, cyanocobalamin 0.03, niacin 30, biotin 0.1, choline chloride 350, folic acid 1.5, inositol 50, canthaxanthin 10, butylated hydroxytoluene 1.5, butylated hydroxyanisole 1.5, α-cellulose 322.1.

^*9*^ The mineral complex (g/kg premix): CaHPO_4_.2H_2_O 295.5, Ca(H_2_PO_4_)_2_.H_2_O 217, NaHCO_3_ 94.5, Na_2_SeO_3_·5H_2_O 0.011, KCl 100, NaCl 172.4, KI 0.2, MgCl_2_ 63.7, MgSO_4_.7H_2_O 70.32, MnSO_4_.H_2_O 1.52, FeSO_4_.7H_2_O 12.41, CuSO_4_.5H_2_O 0.4, ZnSO_4_.7H_2_O 10.

**Table 2:** Fatty acid composition (mg/g of dry matter) of the control diet (FO) and the linseed oil diet (LO).

Fatty acids	FO	LO
14:0	3.0	0.1
16:0	11.5	6.5
18:0	2.4	3.6
16:1n-7	4.3	0.1
18:1n-7	3.1	0.9
18:1n-9	12.9	15.8
20:1n-9	6.2	0.0
18:2n-6	7.9	17.2
18:3n-6	0.1	0.0
20:2n-6	0.2	0.0
20:3n-6	0.1	0.0
20:4n-6	0.4	0.1
22:4n-6	0.0	0.0
22:5n-6	0.2	0.0
18:3n-3	1.1	38.6
18:4n-3	1.5	0.0
20:3n-3	0.2	0.0
20:4n-3	0.7	0.0
20:5n-3	6.6	0.3
22:5n-3	1	0.0
22:6n-3	10	0.8
Total	74.6	84.5
Ʃ SFA ^1^	17.2	10.5
Ʃ MUFA ^2^	27.4	16.9
Ʃ C18 n-6 PUFA ^3^	8.0	17.2
Ʃ n-6 LC-PUFA ^4^	0.9	0.2
Ʃ C18 n-3 PUFA ^5^	2.6	38.6
Ʃ n-3 LC-PUFA ^6^	18.5	1.2
n-3/n-6 ^7^	2.4	2.3
n-3 LC-PUFA /n-6 LC-PUFA ^8^	20.0	6.5

^1^ Sum of saturated fatty acids, includes 20:0, 22:0 and 24:0.

^2^ Sum of monounsaturated fatty acids, includes 14:1n-5, 22:1n-9 and 24:1n-9.

^3^ Sum of omega-6 polyunsaturated fatty acids with 18 C.

^4^ Sum of omega-6 long chain polyunsaturated fatty acids with 20 C and 22 C.

^5^ Sum of omega-3 polyunsaturated fatty acids with 18C.

^6^ Sum of omega-3 long chain polyunsaturated fatty acids with 20 C, 22 C and 24 C.

^7^ Ratio of total omega-3 polyunsaturated fatty acids to total omega-6 polyunsaturated fatty acids.

^8^ Ratio of omega-3 long chain polyunsaturated fatty acids to omega-6 long chain polyunsaturated fatty acids.

### Fish and facilities

A feeding trial firstly provided data on growth performance, body proximate composition, fatty acid composition, and gene expression and desaturase activity in tissues of fish reared at an optimal growth temperature of 15°C or an increased temperature of 19°C and fed FO or LO. In addition, a digestibility trial was conducted at 19°C to evaluate the apparent digestibility of fatty acids in the experimental diets in order to apply the whole body fatty acid balance method.

Prior to the experiments, fish of domesticated origin (Pisciculture d’Hatrival, Hatrival, Belgium) were acclimatised at 12 ± 1°C and fed a commercial diet. The 60-day feeding trial was performed with rainbow trout of an initial mean weight of 8 g. After 48 h of feed deprivation, fish were randomly distributed among twenty tanks (55 l water volume) at a density of 40 fish per tank. Three additional tanks were used as initial condition and fish were anaesthetised directly after fish loading, weighed, dissected if necessary and kept frozen (-20°C) until homogenisation. Ten tanks were set at the optimal water temperature of 15.0 ± 0.9°C and ten tanks at the increased water temperature of 19.0 ± 0.5°C. Five tanks at each temperature were allocated to each dietary treatment (*n* = 5 by experimental condition). The experiment duration was chosen to ensure a minimum tripling in body weight for all groups. Feeding was carried out by hand twice daily (09.00 and 17.00) to apparent satiation. The water was supplied at a 1 l/min flow and temperature was checked daily. Fish were subjected to a 12:12 h light:dark cycle photoperiod. Mortalities were recorded daily and dead fish removed. At the end of the feeding trial and after 48 h of feed deprivation, ten fish were weighed and stored together and seven more were weighed separately and dissected in order to collect the liver and intestine. Initial and final fish were freeze-dried, homogenised (Retsch, Haan, Allemagne) and stored at -20°C until analysis whereas liver and intestine were directly stored at -80°C after having been frozen in liquid nitrogen. The remaining fish from the feeding trial were brought together in one tank with a temperature of 12 ± 1°C and fed a commercial diet until the digestibility trial. The digestibility trial was performed on rainbow trout with an initial mean weight of 187 g. Each dietary treatment was applied to three circular tanks (130 l water volume) with 5.00 ± 0.05 kg of initial fresh fish body weight. The water temperature was maintained at 19.0 ± 0.3°C throughout the trial. Fish were subjected at a 12:12 h light:dark cycle photoperiod. After an adaptation feeding period of 3 days, the experiment was initiated and lasted 24 days in order to accumulate sufficient faeces. Fish were fed manually twice daily (09.00 and 17.00) to apparent satiation whilst avoiding any undesirable mixing of feed and faeces. The faeces were collected continuously through a rotating automatic faeces collector system [43]. The collected faeces were freeze-dried, homogenised and stored at -20°C until further analysis.

### Chemical analyses

The DM, crude ash, crude protein and crude lipid were analysed following analytical methods from the Association of Official Analytical Chemists [44] if not specified below. Briefly, DM and crude ash were measured by drying at 105°C for 16 h followed by an incineration at 550°C for 16 h. Crude protein was determined for diets after acid digestion with the Kjeldahl method (N × 6.25). Crude lipid was evaluated using diethyl ether extraction according to the Soxhlet method. Crude protein content of whole fish was determined as follows: crude protein (% DM) = 100 –crude ash (% DM)–crude lipid (% DM). Gross energy of diets was approximated as follows: gross energy (kJ/100 g DM) = crude protein (% DM) × 23.6 + crude lipid (% DM) × 39.5 + carbohydrates (% DM) × 17.2; where carbohydrates (% DM) = 100—crude ash (% DM)—crude protein (% DM)—crude lipid (% DM) [20]. The chromium III (trivalent) concentration in diets and faeces was determined as described in [45]. Briefly, the protocol consisted in an acid digestion followed by an oxidation step and a spectrophotometric measurement (Cecil Instruments, Cambridge, UK) at 350 nm.

### Performance parameters and fatty acid metabolism computation

Standard formulae were used to assess growth performance, feed utilisation and biometrical parameters throughout the feeding trial. These included initial and final weight, daily growth coefficient (DGC) expressed in (g^1/3^/ day) x 1000 and calculated as follows: [(final body weight)^1/3^ –(initial body weight)^1/3^] x 1000/ (number of feeding days), thermal growth coefficient calculated as follows: [(final body weight) 1/3 –(initial body weight) ^1/3^ / temperature degree-days] x 1000, voluntary feed intake in g of dry feed/fish calculated as follows: $\sum_{i=0}^{t}$ (dry feed ingested at day *i* / number of fish at day *i*), t = number of feeding days, feed intake in %/day calculated as follows: [feed intake in g of dry feed/fish / (mean body weight x number of feeding days)] x 100, feed efficiency expressed in g/g of dry feed and calculated as follows: (final body weight–initial body weight) / feed intake in g of dry feed/fish, protein efficiency ratio (PER) calculated as follows: (final body weight–initial body weight) / (nitrogen intake), nitrogen retention efficiency (NRE) expressed in % and calculated as follows: [(final body nitrogen–initial body nitrogen) / (nitrogen intake)] x 100, lipid efficiency ratio (LER) calculated as follows: (final body weight–initial body weight) / (lipid intake), lipid retention efficiency (LRE) expressed in % and calculated as follows: (final body lipid–initial body lipid) / (lipid intake) x 100, hepatosomatic index expressed in % and calculated as follows: (liver weight / body weight) x 100, intestinosomatic index expressed in % and calculated as follows: (intestine weight / body weight) x 100, and liposomatic index expressed in % and calculated as follows: (perivisceral lipid weight / body weight) x 100.

The estimation of the apparent *in vivo* fatty acid metabolism was calculated via the implementation of the whole body fatty acid balance method [40], with subsequent developments [41, 46]. Briefly, data relative to growth performance and feed intake, dietary and whole body fatty acid composition, and fatty acid digestibility were used in the computations required for the implementation of the method. The apparent fatty acid digestibility was assessed using the standard formula: 100 –[100 × (Cr_2_O_3_ in diet (mg/g DM)) / (Cr_2_O_3_ in faeces (mg/g DM)) × (fatty acid in faeces (mg/g DM)) / (fatty acid in diet (mg/g DM))]. The net appearance/disappearance of each fatty acid was determined as the difference between total fatty acid gain (= final fatty acid content—initial fatty acid content) and the net fatty acid intake (= total fatty acid intake—fatty acid egestion in faeces). The subsequent step involved a series of backwards computations along all the fatty acid bioconversion pathways [n-3 and n-6 polyunsaturated fatty acids (PUFA), saturated fatty acid (SFA) and monounsaturated fatty acids (MUFA)], as previously described in details [40, 41, 46]. Thanks to these calculations, the fate of each individual fatty acid towards bioconversion, oxidation or deposition, was therefore determined. Final results are reported as apparent *in vivo* enzymatic activity expressed as nmol per g of fish per day.

### Fatty acid profile determination

For fatty acid profile determination of diets and whole fish, lipids were extracted following the method of Folch *et al*. [47] subsequently modified [46, 48]. Briefly, lipids of 1 g of dried sample were extracted by a mixture of chloroform/methanol (2:1, *v*:*v*) (VWR chemicals, Radnor, PA, USA). Tridecanoic acid (Sigma-Aldrich) was used as internal standard for lipid quantification. The extracted fatty acids were converted into fatty acid methyl esters (FAME) via methylation in alkaline condition (KOH in methanol, 0.1 M, at 70°C for 60 min) and then in acid condition (HCl in methanol, 1.2 M, at 70°C for 20 min) and FAME subsequently separated by gas chromatography. The GC Trace (Thermo Scientific, Milan, Italy) was equipped with an RT2560 capillary column (100 m × 0.25 mm internal diameter, 0.2 μm film thickness) (Restek, Bellefonte, PA, USA), an automatic injector and a flame ionisation detector kept at a constant temperature of 255°C. The system used hydrogen as carrier gas at an operating pressure of 200 kPa. The oven temperature program was as follows: an initial temperature of 80°C, which progressively increased at 25°C/min up to 175°C, a holding temperature of 175°C during 25 min followed by an increase at 10°C/min up to 205°C, a holding temperature of 205°C during 4 min followed by an increase at 10 C/min up to 225°C and a holding temperature of 225°C during 20 min. Each peak was identified by comparison of retention times with those for pure methyl ester standards (Larodan and Nu-Check Prep, Elysian, USA). Data processing was via ChromQuest software 5.0 (Thermo Finnigan, Milan, Italy). The final results are expressed in mg/g of dry matter.

### Tissue RNA extraction and quantitative realtime PCR (qPCR)

Gene expression was determined as previously described in Geay *et al*. [49]. Total RNA from approximately 100 mg of liver and intestine tissues was extracted using Extract-All® reagent (Eurobio, Courtaboeuf, France) followed by phase separation with chloroform and then precipitated with isopropanol. Based on the nucleic acid concentration measured by spectrophotometry (Nanodrop 2000c, NanoDrop Technologies, Wilmington, DE, USA), 20 μg of RNA was treated with the RTS DNase^TM^ kit (MO BIO Laboratories, Carlsbad, CA, USA) in order to avoid genomic DNA contamination. RNA (1 μg) was then reverse-transcribed using the iScript cDNA Synthesis Kit (Bio-Rad Laboratories, Hercules, CA, USA). The relative expression of the *fatty acid desaturase 2* (f*ads2)* and *elongase 5* (*elovl5)* genes was measured by real-time quantitative reverse transcription polymerase chain reaction (RT-qPCR). PCR primers were designed according to the rainbow trout cDNA sequences for *fads2* and e*lovl5* (Table 3). Amplification of the correct cDNA was confirmed by sequencing. The elongation factor 1-α (*EF1α*) and *β-actin* gene expressions were verified not to be regulated by dietary treatment and temperature and were therefore used as reference genes to normalise the data (Table 3). Amplification of cDNA was carried out using the iQ^TM^SYBR® Green Supermix (Bio-Rad Laboratories). Thermal cycling and fluorescence detection were conducted in a StepOnePlus Real-Time PCR System (Life technologies, Carlsbad, CA, USA) under the following conditions: 10 min of initial denaturation at 95°C, 40 cycles of 15 s at 95°C and 1 min at 60°C. After each run, amplification of single amplicon was confirmed by analysing the melt curve for each sample analysed in triplicate. Standard curves were performed for each primer set and primer efficiency (E) calculated as E = 10^(-1/slope)^. The relative expression of f*ads2* and e*lovl5* was obtained by normalising the mRNA levels of both genes to the geometric mean of *EF1α* and *β-actin* calculated with the relative standard curve method [50].

**Table 3:** Primers used for gene expression determination by quantitative real-time RT-PCR.

Gene	Primer	Sequence primer (5’-3’)	GenBank Acc. No.	Amplicon length
*fads2*	Forward	CGTCCTGGGAGACAAACAGC	AF301910	256 bp
	Reverse	CTGATCAATGCTACGGAGCC		
*elovl5*	Forward	CTATGGGCTCTCTGCTGTCC	AY605100	107 bp
	Reverse	TATCGTCTGGGACATGGTCA		
*Β-actin*	Forward	TTCAACCCTGCCATGT	AB196465	59 bp
	Reverse	ACGGCCAGAGGCGTACAG		
*EF1α*	Forward	ACCCTCCTCTTGGTCGTTTC	AF498320	64 bp
	Reverse	TGATGACACCAACAGCAACA		

*fads2*, fatty acid desaturase 2; *elovl5*, elongase 5; *EF1α*, elongation factor 1 alpha.

### Δ6 Desaturation activity

The Δ6 desaturase enzymatic activity was performed on liver and intestine microsomes as previously described [49]. The tissue was homogenised in sucrose phosphate buffer (0.04 M, pH 7.4) containing 0.25 M sucrose, 0.15 M KCl, 40 mM KF and 1 mM *N*-acetylcysteine and then centrifuged at 25 000 g for 15 min in order to remove the fat upper layer and to collect the supernatant. The supernatant was then centrifuged at 105 000 g for 60 min at 4°C and the microsomal pellet was collected. The protein concentration of the microsomal pellet was determined using the Bio-Rad protein assay (Bio-Rad Laboratories) according to the Bradford dye-binding method [51]. Microsomes were incubated with 0.25 μCi of [1-^14^C]18:3n-3 (Perkin Elmer, Waltham, MA, USA), added as a complex with fatty acid free- bovine serum albumin in phosphate-buffered saline, at 20°C for 1 h. The reaction was stopped with the first step of lipid extraction consisting in addition of chloroform/methanol (2:1, *v*:*v*) containing 0.01% (w/v) butylated hydroxytoluene (BHT) as antioxidant. Lipids were transmethylated to FAME by acid-catalysed transesterification using toluene and 1% H_2_SO_4_ in methanol overnight at 50°C. FAME were dissolved in 100 μl isohexane and applied as streaks on thin-layer chromatography (TLC) plates (Merck, Darmstadt, Germany) previously coated with 2 g silver nitrate in 20 ml acetonitrile and activated at 110°C for 30 min. The TLC plates were fully developed in toluene/acetonitrile (95:5, *v*:*v*). Autoradiography was then performed with a Kodak MR2 film for seven days at room temperature. The area of silica containing the [1-^14^C]18:4n-3 product was scraped into a scintillation mini-vial containing 2.5 ml of scintillation fluid (Meridian Biotechnologies, Epsom, UK) and the radioactivity was determined in a TRI-CARB 2000CA scintillation counter (United Technologies Packard, UK). Results were corrected for counting efficiency and quenching of ^14^C under these conditions and were expressed as pmol of [1-14C]18:4n-3 by hour and mg of microsomal protein. For technical reasons, no results could be obtained for the microsomes derived from liver samples of fish held at 15°C.

### Statistical analysis

All the data are presented as mean ± SEM (*n* = 4, 5 or 35, as stated). Effects of water temperature (T), dietary treatment (D), and water temperature × dietary treatment interaction (T × D) were analysed by a two-way analysis of variance (ANOVA), followed by Tukey’s (parametric) or Student’s (nonparametric with α = 0.08%) *post hoc* test in order to determine significant differences between conditions. Previously to statistical analysis, data were transformed with natural logarithm or square root if identified as non-homogenous (Levene’s test) to meet the assumptions for statistical methods. Results of the two-way ANOVA test are reported in the Results section as: ns (not significant, P > 0.05), * (P < 0.05) or ** (P < 0.01). Statistical analysis was computed using JMP® Pro 11 (SAS, Cary, NC, USA).

## Results

Each result section is presented considering firstly the temperature impact, then the dietary treatment impact, and finally the temperature and dietary treatment interaction impact if relevant. Two-way ANOVA and *post hoc* tests were used to compare the results and information is given in case of contradictory statistical results between both statistical tests.

### Fish growth performance

The experimental conditions were readily accepted by fish and body weight increased by a minimum factor of six, as observed in Table 4. The mean mortality rate throughout the feeding trial was less than 0.1% per day and was unrelated to the temperature or the diet.

**Table 4:** Growth performance, feed utilisation and biometrical parameters of rainbow trout reared at 15°C or 19°C on a control diet (FO) or a linseed oil diet (LO) over 60 feeding days.

	15°C		19°C				
	FO	LO	FO	LO	T	D	T × D
*Growth performance and feed utilisation parameters *							
Initial weight (g/fish)	8.17 ± 0.04	8.16 ± 0.08	7.92 ± 0.12	8.20 ± 0.04	ns	ns	ns
Final weight (g/fish)	54.35 ± 1.62	51.49 ± 1.70	53.41 ± 1.48	48.60 ± 1.11	ns	*	ns
DGC ((g^1/3^/day) x 1000)	29.54 ± 0.60 ^a^	28.43 ± 0.61 ^ab^	29.52 ± 0.50 ^a^	27.20 ± 0.51 ^b^	ns	**	ns
Thermal growth coefficient	1.97 ± 0.04 ^a^	1.90 ± 0.04 ^a^	1.55 ± 0.03 ^b^	1.43 ± 0.03 ^c^	**	**	ns
Feed intake in g of dry feed/fish	39.23 ± 1.13	36.81 ± 0.94	40.55 ± 1.20	37.86 ± 1.36	ns	*	ns
Feed intake in %/ day	2.09 ± 0.05 ^ab^	2.06 ± 0.02 ^b^	2.20 ± 0.03 ^ab^	2.22 ± 0.04 ^a^	**	ns	ns
Feed efficiency (g/g of dry feed)	1.18 ± 0.03 ^a^	1.18 ± 0.02 ^a^	1.12 ± 0.01 ^ab^	1.07 ± 0.01 ^b^	**	ns	ns
PER	2.39 ± 0.06 ^a^	2.36 ± 0.04 ^a^	2.27 ± 0.03 ^ab^	2.14 ± 0.03 ^b^	**	ns	ns
NRE (%)	37.13 ± 1.01 ^ab^	38.39 ± 0.69 ^a^	37.74 ± 0.55 ^a^	34.53 ± 0.15 ^b^	*	ns	**
LER	10.91 ± 0.28 ^a^	10.20 ± 0.19 ^a^	10.39 ± 0.12 ^a^	9.27 ± 0.11 ^b^	**	**	ns
LRE (%)	96.60 ± 3.13 ^a^	80.24 ± 2.11 ^bc^	86.22 ± 1.34 ^b^	76.17 ± 1.32 ^c^	**	**	ns
*Biometrical parameters *							
Hepatosomatic index (%)	1.39 ± 0.04 ^a^	1.48 ± 0.03 ^a^	1.04 ± 0.03 ^b^	1.10 ± 0.03 ^b^	**	*	ns
Intestinosomatic index (%)	3.23 ± 0.08 ^a^	3.36 ± 0.1 ^a^	2.76 ± 0.08 ^b^	2.84 ± 0.07 ^b^	**	ns	ns
Liposomatic index (%)	1.51 ± 0.08	1.7 ± 0.09	1.49 ± 0.09	1.67 ± 0.08	ns	*	ns

Mean values (± SEM) within a row with no common superscript letter (^a, ab, b, bc, c^) are significantly different (Tukey’s *post hoc* test on log transformed values). P values relative to two-way ANOVA are reported in the last three columns of the table (T, water temperature effect; D, dietary treatment effect; T × D, water temperature × dietary treatment interaction; ns *P* > 0.05

* *P* < 0.05

** *P* < 0.01

*n* = 5 or hepatosomatic, intestinosomatic and liposomatic indeces: *n* = 35). DGC, daily growth coefficient; PER, protein efficiency ratio; NRE, nitrogen retention efficiency; LER, lipid efficiency ratio; LRE, lipid retention efficiency.

#### Temperature impact

The increased temperature of 19°C induced no impact on fish weight and DGC but decreased the thermal growth coefficient for both dietary conditions (*P* < 0.01). Increased feed intake, expressed in % per day, was observed with increased temperature (*P* < 0.01), as highlighted by the *post hoc* statistical test, especially with fish fed LO. In contrast, the feed efficiency decreased for this condition (*P* < 0.01). More precisely, the temperature increase significantly reduced the feed conversion efficiency ratio and the retention efficiency of dietary proteins (PER and NRE, respectively) and lipids (LER and LRE, respectively) of fish fed LO, although the reduction of LRE was only shown by the ANOVA test. With both dietary treatments, the hepatosomatic and intestinosomatic indeces decreased with the temperature increase (*P* < 0.01).

#### Diet impact

Considering fish held at 19°C, growth was negatively impacted by the reduced DGC and thermal growth coefficient (*P* < 0.01) for fish fed LO compared to FO. In contrast, fish growth was not impacted by dietary treatment in fish held at 15°C. Feed intake, expressed in % per day, and feed efficiency were unrelated to diet (*P* > 0.05). However, at the increased temperature of 19°C, NRE and dietary lipid conversion and retention, LER and LRE respectively, decreased in fish fed LO compared to fish fed FO. Slight increases in the hepatosomatic and liposomatic indeces were recorded in fish fed LO (*P* < 0.05), although these were not highlighted by the *post hoc* test.

### Fish proximate composition

#### Temperature impact

No impact of temperature was observed on whole fish proximate composition, as observed in Table 5, although ANOVA revealed a temperature effect on crude protein content.

**Table 5:** Initial and final proximate composition (mg/g of wet matter) of rainbow trout subjected to a feeding trial at 15°C or 19°C with a control diet (FO) or a linseed oil diet (LO).

		15°C		19°C				
	Initial	FO	LO	FO	LO	T	D	T × D
Dry matter	241.7 ± 5.1	264.4 ± 1.3	261.4 ± 1.4	269.7 ± 1.6	264.4 ± 3.5	ns	ns	ns
Crude ash	21.0 ± 0.7	23.8 ± 0.4	23.3 ± 0.5	24.7 ± 1.1	24.7 ± 0.6	ns	ns	ns
Crude lipid	79.3 ± 0.8	87.1 ± 1.5 ^a^	78.8 ± 0.9 ^b^	82.4 ± 0.9 ^ab^	81.8 ± 1.9 ^ab^	ns	**	*
Crude protein	141.3 ± 3.7	153.5 ± 0.7	159.3 ± 0.4	162.5 ± 2.2	157.8 ± 1.7	*	ns	**

Mean values (± SEM) within a row with no common superscript letter (^a, ab, b^) are significantly different (Tukey’s *post hoc* test on log transformed final condition values). P values relative to two-way ANOVA are reported in the last three columns of the table (T, water temperature effect; D, dietary treatment effect; T × D, water temperature × dietary treatment interaction; ns *P* > 0.05

* *P* < 0.05

** *P* < 0.01; *n* = 5).

#### Diet impact

The dietary treatment did not affect whole fish proximate composition, with the exception of reduced crude lipid content of fish fed LO compared to fish fed FO at 15°C (*P* < 0.01).

### Whole body fatty acid composition

#### Temperature impact

The fatty acid composition of whole trout is presented in Table 6. No effect of temperature was recorded on the SFA and MUFA contents. Regarding fish fed LO, the temperature increase induced increased C18 n-6 PUFA content (mainly LA) and a slight decrease in n-6 LC-PUFA content (*P* < 0.05). Regarding the n-3 family, more C18 n-3 PUFA and less n-3 LC-PUFA were observed at increased temperature. This last effect was however detected by the ANOVA test (*P* < 0.01) but not by the *post hoc* test. The ALA content was higher for fish raised at 19°C compared to 15°C, especially with fish fed LO, whereas the ANOVA statistical test highlighted the opposite effect on 20:4n-3 and EPA contents (*P* < 0.01). Concerning fish fed FO, lower DHA content was recorded with increased temperature. The n-3/n-6 ratio was slightly reduced with the temperature increase (*P* < 0.05) whereas the n-3 LC-PUFA /n-6 LC-PUFA ratio was not impacted.

**Table 6:** Initial and final whole body fatty acid profile (mg/g of dry matter) of rainbow trout subjected to a feeding trial at 15°C or 19°C with a control diet (FO) or a linseed oil diet (LO).

		15°C		19°C				
Fatty acids	Initial	FO	LO	FO	LO	T	D	T × D
18:2n-6	18.77 ± 0.50	19.85 ± 0.22 ^c^	29.89 ± 0.61 ^b^	19.71 ± 0.20 ^c^	32.42 ± 0.74 ^a^	*	**	*
18:3n-6	0.66 ± 0.01	0.47 ± 0.01 ^b^	0.84 ± 0.02 ^a^	0.40 ± 0.01 ^c^	0.80 ± 0.02 ^a^	**	**	Ns
20:2n-6	1.08 ± 0.04	1.48 ± 0.02	1.62 ± 0.08	1.38 ± 0.02	1.66 ± 0.07	ns	**	Ns
20:3n-6	0.89 ± 0.03	0.93 ± 0.01 ^b^	1.47 ± 0.05 ^a^	0.78 ± 0.01 ^c^	1.30 ± 0.03 ^a^	**	**	Ns
20:4n-6	2.45 ± 0.07	1.27 ± 0.02 ^a^	1.07 ± 0.03 ^b^	1.23 ± 0.02 ^a^	1.00 ± 0.03 ^b^	ns	**	Ns
22:4n-6	0.24 ± 0.00	0.13 ± 0.01 ^a^	0.1 ± 0.00 ^b^	0.13 ± 0.01 ^a^	0.09 ± 0.01 ^b^	ns	**	Ns
22:5n-6	0.72 ± 0.06	0.44 ± 0.03	0.36 ± 0.04	0.45 ± 0.02	0.37 ± 0.02	ns	**	Ns
18:3n-3	2.94 ± 0.02	2.28 ± 0.08 ^c^	36.81 ± 1.02 ^b^	2.67 ± 0.10 ^c^	40.39 ± 1.10 ^a^	**	**	Ns
18:4n-3	4.07 ± 0.04	1.95 ± 0.03 ^b^	4.47 ± 0.06 ^a^	1.91 ± 0.02 ^b^	4.47 ± 0.09 ^a^	ns	**	Ns
20:3n-3	0.48 ± 0.03	0.45 ± 0.03 ^b^	2.83 ± 0.13 ^a^	0.51 ± 0.03 ^b^	2.87 ± 0.14 ^a^	ns	**	Ns
20:4n-3	2.87 ± 0.09	1.48 ± 0.04 ^b^	2.49 ± 0.18 ^a^	1.30 ± 0.01 ^b^	2.13 ± 0.08 ^a^	**	**	Ns
20:5n-3	21.17 ± 0.34	7.75 ± 0.14 ^a^	4.42 ± 0.13 ^b^	7.34 ± 0.16 ^a^	4.11 ± 0.04 ^b^	**	**	Ns
22:5n-3	6.44 ± 0.21	2.57 ± 0.05 ^a^	1.56 ± 0.09 ^b^	2.59 ± 0.05 ^a^	1.63 ± 0.01 ^b^	ns	**	Ns
24:5n-3	1.11 ± 0.06	0.62 ± 0.04 ^a^	0.22 ± 0.03 ^b^	0.56 ± 0.01 ^a^	0.23 ± 0.01 ^b^	ns	**	Ns
24:6n-3	1.52 ± 0.02	0.85 ± 0.03 ^a^	0.46 ± 0.01 ^b^	0.85 ± 0.02 ^a^	0.51 ± 0.02 ^b^	ns	**	Ns
22:6n-3	41.61 ± 0.98	25.03 ± 0.35 ^a^	14.65 ± 0.33 ^c^	23.73 ± 0.23 ^b^	14.02 ± 0.21 ^c^	**	**	Ns
Ʃ SFA ^1^	81.77 ± 1.70	80.98 ± 0.55 ^a^	63.42 ± 1.70 ^b^	76.75 ± 1.08 ^a^	67.68 ± 2.04 ^b^	ns	**	*
Ʃ MUFA ^2^	81.77 ± 1.54	122.48 ± 0.38 ^a^	94.88 ± 3.11 ^c^	112.18 ± 1.62 ^b^	98.68 ± 3.29 ^c^	ns	**	*
Ʃ C18 n-6 PUFA ^3^	19.42 ± 0.51	20.32 ± 0.22 ^c^	30.73 ± 0.61 ^b^	20.11 ± 0.20 ^c^	33.22 ± 0.76 ^a^	*	**	*
Ʃ n-6 LC-PUFA ^4^	5.38 ± 0.18	4.24 ± 0.06 ^ab^	4.62 ± 0.09 ^a^	3.97 ± 0.03 ^b^	4.42 ± 0.14 ^ab^	*	**	Ns
Ʃ C18 n-3 PUFA ^5^	7.01 ± 0.06	4.23 ± 0.09 ^c^	41.28 ± 1.06 ^b^	4.58 ± 0.10 ^c^	44.86 ± 1.15 ^a^	**	**	Ns
Ʃ n-3 LC-PUFA ^6^	75.22 ± 1.71	38.75 ± 0.51 ^a^	26.62 ± 0.63 ^b^	36.87 ± 0.33 ^a^	25.49 ± 0.40 ^b^	**	**	Ns
n-3/n-6 ^7^	3.32 ± 0.02	1.75 ± 0.00 ^b^	1.92 ± 0.03 ^a^	1.72 ± 0.02 ^b^	1.87 ± 0.01 ^a^	*	**	Ns
n-3 LC-PUFA /n-6 LC-PUFA ^8^	14.00 ± 0.18	9.14 ± 0.07 ^a^	5.77 ± 0.22 ^b^	9.29 ± 0.11 ^a^	5.78 ± 0.11 ^b^	ns	**	Ns
Total	270.66 ± 5.58	271.00 ± 1.32 ^a^	261.55 ± 5.59 ^a^	254.49 ± 2.76 ^a^	274.36 ± 7.36 ^a^	ns	ns	**

Mean values (± SEM) within a row with no common superscript letter (^a, ab, b, c^) are significantly different (Tukey’s (parametric) or Student’s (nonparametric with α = 0.8%) *post hoc* tests on square root transformed final condition values). P values relative to two-way ANOVA are reported in the last three columns of the table (T, water temperature effect; D, dietary treatment effect; T × D, water temperature × dietary treatment interaction

ns *P* > 0.05

* *P* < 0.05

** *P* < 0.01; *n* = 5 except for LO at 15°C for which *n* = 4).

^1^ Sum of saturated fatty acids, includes 20:0, 22:0 and 24:0.

^2^ Sum of monounsaturated fatty acids, includes 14:1n-5, 22:1n-9 and 24:1n-9.

^3^ Sum of omega-6 polyunsaturated fatty acids with 18 C.

^4^ Sum of omega-6 long chain polyunsaturated fatty acids with 20 C and 22 C.

^5^ Sum of omega-3 polyunsaturated fatty acids with 18C.

^6^ Sum of omega-3 long chain polyunsaturated fatty acids with 20 C, 22 C and 24 C.

^7^ Ratio of total omega-3 polyunsaturated fatty acids to total omega-6 polyunsaturated fatty acids.

^8^ Ratio of omega-3 long chain polyunsaturated fatty acids to omega-6 long chain polyunsaturated fatty acids.

#### Diet impact

Decreased SFA and MUFA contents were observed for fish fed LO (Table 6). In contrast, an increase in LA and ALA and their desaturation products, 18:3n-6 and 18:4n-3 respectively, was observed with the replacement of FO by LO. The n-6 LC-PUFA content slightly increased upon feeding fish with LO mainly due to a significant increase in 20:3n-6 (*P* < 0.01), although the n-6 LC-PUFA, ARA and 22:4n-6, decreased. The level of n-3 LC-PUFA was reduced in fish fed LO (*P* < 0.01). This was due to decreased contents of EPA, 22:5n-3 and DHA. Amounts of 20:3n-3 and 20:4n-3 increased however in the presence of the ALA precursor in fish fed LO. Overall, the lipid source replacement led to an increased n-3/n-6 ratio whereas the n-3 LC-PUFA /n-6 LC-PUFA ratio decreased for fish fed LO (*P* < 0.01).

#### Temperature × Diet impact

A temperature × diet interaction was observed on C18 n-6 PUFA, specially the LA content, as no effect of temperature was observed in fish fed FO whereas fish fed LO exhibited an increase in these contents with increased temperature (*P* < 0.05).

### Fatty acid appearance and disappearance

#### Temperature impact

As observed in Table 7, the n-6 pathway bioconversion capacity seemed to be affected by the temperature increase with reduced appearances of some intermediates such as 18:3n-6, 20:3n-6 and ARA (*P* < 0.05). The lower appearances were nevertheless not significant as highlighted by the *post hoc* tests. Regarding the n-3 family, the ALA disappearance was higher in fish fed LO at increased temperature (*P* < 0.05). While 18:4n-3 and 20:3n-3 were not impacted by temperature, 20:4n-3, EPA and DHA appearances were reduced at increased temperature, as shown by ANOVA (*P* < 0.05).

**Table 7:** Appearance and disappearance of fatty acids deduced by the whole body fatty acid balance method (nmol per g of fish per day) of rainbow trout reared at 15°C or 19°C on a control diet (FO) or a linseed oil diet (LO).

	15°C		19°C				
Fatty acids	FO	LO	FO	LO	T	D	T × D
18:2n-6	-61.2 ± 18.4 ^a^	-471.5 ± 33.2 ^b^	-82.0 ± 7.0 ^a^	-512.5 ± 19.1 ^b^	ns	**	ns
18:3n-6	3.5 ± 0.3 ^b^	20.0 ± 0.7 ^a^	1.6 ± 0.2 ^c^	18.9 ± 0.6 ^a^	**	**	ns
20:2n-6	18.9 ± 0.9 ^b^	34.5 ± 2.3 ^a^	16.6 ± 0.5 ^b^	35.2 ± 2.2 ^a^	ns	**	ns
20:3n-6	16.3 ± 0.5 ^b^	30.6 ± 0.8 ^a^	12.8 ± 0.3 ^c^	26.1 ± 1.2 ^a^	**	**	ns
20:4n-6	-3.3 ± 1.1 ^b^	14.0 ± 1.2 ^a^	-4.9 ± 0.7 ^b^	11.3 ± 1.0 ^a^	*	**	ns
22:4n-6	-0.8 ± 0.2 ^a^	-1.2 ± 0.1 ^ab^	-1.1 ± 0.1 ^ab^	-1.7 ± 0.1 ^b^	*	**	ns
22:5n-6	-1.3 ± 0.8 ^b^	5.5 ± 1.0 ^a^	-1.2 ± 0.4 ^b^	5.5 ± 0.5 ^a^	ns	**	ns
18:3n-3	-22.6 ± 4.0 ^a^	-1785.8 ± 53.1 ^b^	-14.4 ± 2.4 ^a^	-1930.8 ± 41.2 ^b^	*	**	*
18:4n-3	-66.7 ± 3.0 ^b^	104.7 ± 3.1 ^a^	-72.0 ± 1.6 ^b^	103.6 ± 3.1 ^a^	ns	**	ns
20:3n-3	-2.3 ± 0.9 ^b^	65.6 ± 3.7 ^a^	-1.3 ± 0.9 ^b^	66.5 ± 4.0 ^a^	ns	**	ns
20:4n-3	-16.8 ± 2.1 ^b^	50.2 ± 3.6 ^a^	-22.7 ± 0.4 ^b^	40.9 ± 2.4 ^a^	**	**	ns
20:5n-3	-320.1 ± 12.8 ^b^	14.0 ± 2.1 ^a^	-349.2 ± 7.5 ^b^	-0.1 ± 3.2 ^a^	*	**	ns
22:5n-3	-23.6 ± 2.7 ^b^	11.7 ± 1.7 ^a^	-25.1 ± 1.1 ^b^	11.7 ± 1.0 ^a^	ns	**	ns
24:5n-3	9.9 ± 0.8 ^a^	1.2 ± 0.6 ^b^	8.9 ± 0.4 ^a^	1.1 ± 0.2 ^b^	ns	**	ns
24:6n-3	13.8 ± 0.7 ^a^	5.0 ± 0.4 ^b^	14.1 ± 0.3 ^a^	5.7 ± 0.7 ^b^	ns	**	ns
22:6n-3	-154.3 ± 22.3 ^b^	156.1 ± 3.6 ^a^	-204.1 ± 6.7 ^b^	130.1 ± 11.0 ^a^	*	**	ns

Mean values (± SEM) within a row with no common superscript letter (^a, ab, b, c^) are significantly different (Tukey’s (parametric) or Student’s (nonparametric with α = 0.8%) *post hoc* tests). P values relative to two-way ANOVA are reported in the last three columns of the table (T, water temperature effect; D, dietary treatment effect; T × D, water temperature × dietary treatment interaction

ns *P* > 0.05

* *P* < 0.05

** *P* < 0.01

*n* = 5 except for LO at 15°C for which *n* = 4).

#### Diet impact

The LO diet induced higher disappearance of LA and ALA substrates in trout (Table 7), which can be logically related to their dietary large amounts. The n-6 and n-3 bioconversion pathways were positively affected by the dietary lipid source replacement. Indeed, all the n-6 fatty acid intermediates showed higher appearance levels in fish fed LO (*P* < 0.01), with the exception of 22:4n-6. Accordingly, the appearance levels of most of the n-3 fatty acid intermediates increased in fish fed LO. This was the case for all intermediates up to 22:5n-3 (*P* < 0.01), as well as for the highly valuable DHA end product (*P* < 0.01). Only the two C24 intermediates, namely 24:5n-3 and 24:6n-3, showed a decreased appearance level in fish fed LO (*P* < 0.01).

### Apparent *in vivo* fatty acid metabolism

#### Temperature impact

The temperature increase had no impact on either the fatty acid *de novo* synthesis pathway, or on SFA and MUFA β-oxidation and elongation, or on the apparent *in vivo* Δ9 desaturation activity (Table 8). Moreover, in the n-6 bioconversion pathway, no significant effect of temperature was recorded on apparent *in vivo* β-oxidation, elongation and Δ5 desaturation activities involved. The apparent *in vivo* Δ6 desaturation activity was nevertheless reduced with the temperature increase (*P* < 0.01). In contrast, the apparent *in vivo* n-3 bioconversion capacity was consistently affected by temperature in fish fed LO. The n-3 apparent *in vivo* Δ5 and Δ6 desaturation activities decreased with the temperature increase (*P* < 0.05) whereas n-3 fatty acid β-oxidation increased (*P* < 0.01). The apparent *in vivo* n-3 fatty acid elongation activity was also reduced by increased temperature but not significantly (*P* value = 0.06). Considering the sum of the fatty acid products of both n-6 and n-3 pathways, the apparent *in vivo* Δ5 and Δ6 desaturation activities were lower at the increased temperature of 19°C (*P* < 0.05). It is worth noting that the *post hoc* statistical test used for the apparent *in vivo* activity results had to be nonparametric, and was thus less powerful, which may explain why they do not corroborate the ANOVA results related to the significant effects of temperature.

**Table 8:** Fatty acid metabolism (nmol per g of fish per day), deduced by the whole body fatty acid balance method, of rainbow trout reared at 15°C or 19°C on a control diet (FO) or a linseed oil diet (LO).

	15°C		19°C				
	FO	LO	FO	LO	T	D	D × T
**SFA and MUFA** ^**1**^							
*De novo* production	2062 ± 89	1822 ± 201	1634 ± 91	1846 ± 185	ns	ns	ns
β-oxidation	39 ± 14 ^ab^	3 ± 0 ^b^	64 ± 6 ^a^	4 ± 0 ^b^	ns	**	ns
Elongation	5114 ± 217	4449 ± 514	4048 ± 220	4506 ± 465	ns	ns	ns
Δ9 desaturation	1238 ± 48	1082 ± 122	941 ± 52	1064 ± 109	ns	ns	ns
**n-6 PUFA** ^**1**^							
β-oxidation	33 ± 19 ^b^	368 ± 36 ^a^	58 ± 7 ^b^	417 ± 22 ^a^	ns	**	ns
Elongation	35 ± 1 ^b^	93 ± 5 ^a^	29 ± 1 ^b^	86 ± 6 ^a^	ns	**	ns
Δ5 desaturation	0 ± 0 ^b^	18 ± 2 ^a^	0 ± 0 ^b^	15 ± 1 ^a^	ns	**	ns
Δ6 desaturation	20 ± 1 ^b^	74 ± 3 ^a^	14 ± 0 ^b^	66 ± 3 ^a^	**	**	ns
**n-3 PUFA** ^**1**^							
β-oxidation	583 ± 47 ^c^	1377 ± 58 ^b^	666 ± 15 ^c^	1571 ± 36 ^a^	**	**	ns
Elongation	26 ± 1 ^b^	640 ± 16 ^a^	23 ± 1 ^b^	541 ± 46 ^a^	ns	**	ns
Δ5 desaturation	0 ± 0 ^b^	188 ± 8 ^a^	0 ± 0 ^b^	148 ± 16 ^a^	*	**	*
Δ6 desaturation	14 ± 1 ^b^	504 ± 10 ^a^	14 ± 0 ^b^	429 ± 32 ^a^	*	**	*
**n-6 and n-3** ^**1**^							
Elongation	61 ± 2 ^b^	733 ± 15 ^a^	53 ± 1 ^b^	627 ± 51 ^a^	ns	**	ns
**Total**							
Elongation	5175 ± 219	5182 ± 519	4101 ± 220	5133 ± 512	ns	ns	ns
Δ5 desaturation	0 ± 0 ^b^	206 ± 8 ^a^	0 ± 0 ^b^	164 ± 17 ^a^	*	**	*
Δ6 desaturation	34 ± 1 ^b^	579 ± 9 ^a^	28 ± 0 ^b^	494 ± 35 ^a^	*	**	ns

Mean values (± SEM) within a row with no common superscript letter (^a, ab, b, c^) are significantly different (Tukey’s (parametric) or Student’s (nonparametric with α = 0.8%) *post hoc* tests). P values relative to two-way ANOVA are reported in the last three columns of the table (T, water temperature effect; D, dietary treatment effect; T × D, water temperature × dietary treatment interaction

ns *P* > 0.05

* *P* < 0.05

** *P* < 0.01; *n* = 5 except for LO at 15°C for which *n* = 4).

^1^ See Table 2 for abbreviations.

#### Diet impact

The LO feeding reduced the apparent *in vivo* SFA and MUFA β-oxidation (*P* < 0.01) while no impact on the *de novo* production, elongation and Δ9 desaturation was observed. On the contrary, the n-6 and n-3 bioconversion pathways were positively affected when feeding fish LO. For both pathways, the LO diet induced higher apparent *in vivo* β-oxidation, elongation, Δ5 and Δ6 desaturation enzymatic activities compared to FO at both temperatures (*P* < 0.01). Overall, considering the apparent *in vivo* fatty acid metabolism of both pathways, the elongation, Δ5 and Δ6 desaturation activities were increased in fish fed LO (*P* < 0.01).

### *fads2* and *elovl5* gene expression

**Fig 2:**
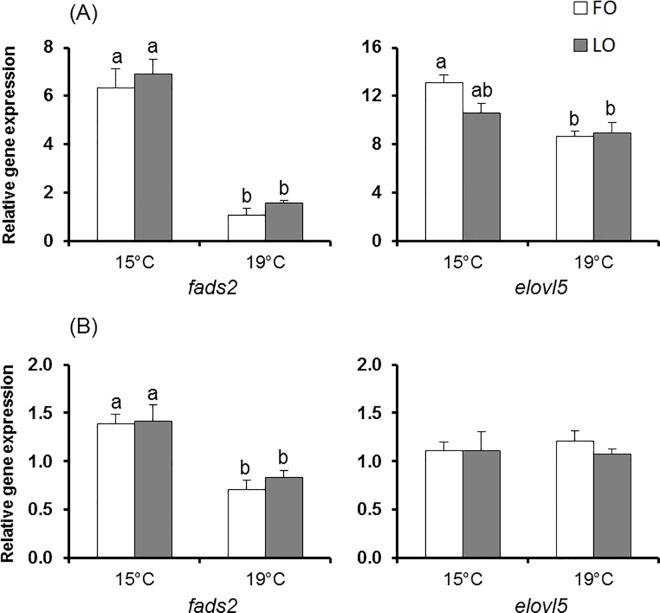
**Effect of the dietary treatment on f*ads2* and e*lovl5* relative expressions in the liver (A) and intestine (B) of rainbow trout reared at two different water temperatures with a fish oil (FO) or a linseed oil (LO) diet.** Results are expressed as relative mean value (± SEM) to geometric mean of *EF1α* and *β-actin* reference gene expressions. On the same graph, data with no common letter (^a, ab, b^) are significantly different (Tukey’s (parametric) or Student’s (nonparametric with α = 0.8%) *post hoc* tests; *n* = 5 except in intestine for which *n* = 4 for LO at 19°C). f*ads2*, fatty acid desaturase 2; *elovl5*, elongase 5; *EF1α*, elongation factor 1α.

The expression of f*ads2* and e*lovl5*, which correspond to the genes of enzymes involved in the first two steps of endogenous fatty acid bioconversion, were higher in the liver compared to the intestine (Fig 2). Moreover, the e*lovl5* expression level was higher than that of f*ads2* in the case of the liver whereas similar expression levels were observed in the intestine.

#### Temperature impact

Irrespective of diet, *fads2* expression was reduced by the increased temperature of 19°C, both in the liver and intestine. In contrast, e*lovl5* expression was negatively impacted by the temperature increase, but only in liver in fish fed FO.

#### Diet impact

The dietary treatment did not affect the expression of f*ads2* or e*lovl5* in either tissue (*P* > 0.05).

### Δ6 Desaturase enzymatic activity

#### Temperature impact

**Fig 3:**
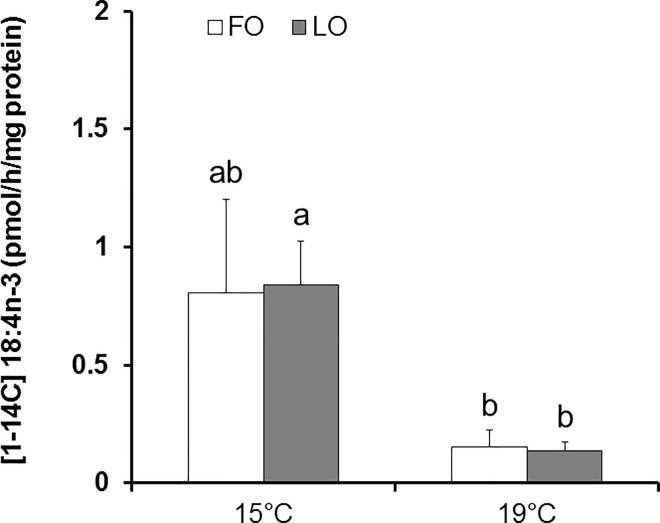
Δ6 Desaturase enzymatic activity (pmol of [1-14C]18:4n-3 by hour and mg of protein) of intestinal microsomes of rainbow trout reared at two different water temperatures on a control diet (FO) or a linseed oil diet (LO). Mean values (± SEM) with no common letter (^a, ab, b^) are significantly different (Tukey’s *post hoc* test on log transformed values; *n* = 5).

The temperature increase induced a six-fold reduction of the Δ6 desaturase enzymatic activity, measured as the rate of desaturation of [1-1^4^C]18:3n-3 to [1-^14^C]18:4n-3 by intestinal microsomes (Fig 3). The difference was however significant only for fish fed LO.

#### Diet impact

The Δ6 desaturase enzymatic activity was not affected by the dietary treatment in intestine, irrespective of temperature (Fig 3). Accordingly, no dietary impact was observed on liver microsomes of fish held at 19°C (3.41 ± 0.8 and 3.93 ± 0.53 pmol × h^-1^ × mg protein^-1^ for fish fed FO and LO, respectively). Interestingly, the Δ6 desaturase enzymatic activity was more than 20-fold higher in liver microsomes than in intestinal microsomes of fish held at 19°C. Unfortunately, due to technical problems with some samples, no results are available for the liver microsomes of fish reared at 15°C.

## Discussion

The present study aimed to evaluate the impact of a 4°C increase in water temperature on the fatty acid bioconversion capacity of rainbow trout fed a LO diet. It had already been reported that this species possesses a good fatty acid bioconversion capacity when fed with plant-derived oils [24, 25, 52–54]. However, less is known about this metabolic capacity in warmer water.

Regarding fish growth performance, the present data indicated that replacing fish oil with linseed oil had nutritional troubles with increased water temperature beyond the limit of the optimal temperature range of rainbow trout (15°C—16°C). In accordance to what was observed in the present study, several earlier studies also described that, in salmonids, increased water temperature can negatively affect fish growth by increasing feed intake [15, 34] but decreasing feed efficiency [55] and hepatosomatic index [34, 55, 56]. Moreover, it has been reported that rainbow trout energy requirements increase, almost linearly, with water temperature [6, 20] and, therefore, induce higher feed intake and lower dietary protein and lipid conversion and retention, as highlighted in the present study. In contrast, the results of published studies are partly contradictory to ours. For example, while feeding adult Atlantic salmon a diet formulated with a blend of fish and rapeseed oils for 56 days, Hevrøy *et al*. [55] found, as here, negative effects of elevated temperature from 14°C to 19°C on growth, feed utilisation, NRE, LRE and hepatosomatic index but feed intake was also negatively impacted. Considering the effect of lipid source replacement on fish growth, the total or substantial replacement of fish oil by linseed oil had already been shown to have no detrimental effect on salmonid growth when fish were held at their optimal growth temperature [18, 23–26, 57]. For fish held at 19°C in the present study, the feed intake and overall feed efficiency were unaffected by dietary treatment but, inconsistently, reduced conversion and retention efficiencies of the dietary lipids (LER and LRE, respectively) were observed. This might be due to the length of the feeding trial, which could have been too short to affect feed efficiency but long enough to allow differences of the LER and LRE coefficients. The reduced LER and LRE observed when the dietary change was imposed at 19°C could indicate a higher fish energy demand in that condition and explain the reduced DGC observed.

In the present study, significantly higher ALA was recorded with the temperature increase in the whole body of fish fed LO. Conversely, the temperature increase slightly reduced the fish n-3 LC-PUFA content. A similar temperature effect on ALA content but an absence of effect on n-3 LC-PUFA content have been reported previously in the whole body of Atlantic salmon fed diets varying in the ARA/EPA ratio from 160 g to around 250 g [34]. Interestingly, the temperature increase slightly increased ALA but highly reduced EPA and DHA in European sea bass (*Dicentrarchus labrax*) fed a rapeseed oil diet [38]. Regarding the n-6 PUFA profile, the temperature increase induced an increase of LA and a slight decrease of n-6 LC-PUFA content. However, no temperature effect was observed on ARA, which is in accordance with data previously reported in European sea bass reared at 22°C or 29°C and fed a rapeseed oil diet [38]. In contrast, increased ARA was reported in Atlantic salmon when water temperature was increased from 10°C to 20°C [34]. As regards the diet impact on fish lipid composition, increased fatty acid bioconversion of LA and ALA in fish fed LO was associated with increased levels of their corresponding products until 20:3n-6 and 20:4n-3, respectively. In contrast, the final n-3 bioconversion products, EPA and DHA, decreased when feeding fish with LO, leading to a reduced n-3 LC-PUFA content with LO. As observed in the present paper and reported by numerous previous studies [23–26, 30, 57], fish fatty acid composition after a feeding trial reflects that of the experimental diet administered. Concerning n-3 LC-PUFA, Tocher *et al*. [58] reported that the fatty acid bioconversion capacity of Atlantic salmon fed a linseed oil diet was increased until 20:4n-3 but ineffective in maintaining similar EPA and DHA contents than fish fed a fish oil diet, despite high dietary ALA, which is consistent with the present data. In order to counteract the EPA and DHA decrease by feeding fish with plant-derived oils, finishing diets, fed at the end of the fish grow-out period and formulated with fish oil, have been investigated to restore the n-3 LC-PUFA content in fish fillet and have shown positive results [18, 23, 24]. Moreover, a study demonstrated that water temperature had no influence on this restoration capacity in rainbow trout held at 15°C or 20°C [59].

The whole body fatty acid balance method previously developed by Turchini *et al*. [40, 41, 46] highlighted firstly a higher disappearance of ALA with the temperature increase. However, this higher disappearance was not correlated with higher appearance of n-3 fatty acid bioconversion products. Regarding the apparent *in vivo* enzymatic activities, the elongases apparent *in vivo* activity was not impacted by temperature considering both the n-6 and n-3 pathways. In contrast, the apparent *in vivo* activities of the Δ5 and Δ6 desaturases were slightly reduced at the increased temperature of 19°C. A recently published study [33] also investigated the impact of a temperature increase on fish lipid metabolism with the use of the whole body fatty acid balance method. This study investigated Atlantic salmon raised at the increased temperature of 20**°**C, in comparison with 10°C, and fed diets varying in ARA/EPA ratio. Consistent with data in the present study, the authors reported a reduced apparent *in vivo* Δ6 desaturation activity and no effect on the Δ9 desaturation activity. In contrast however, no effect on Δ5 desaturation was observed. Regarding the elongation activities, contrasting data were obtained since the apparent *in vivo* activities of elovl5 and elovl2 were not modified and reduced, respectively [33]. A positive effect of linseed oil diets on the rainbow trout fatty acid bioconversion capacity when fish were held at their optimal growth temperature has already been reported [24, 25]. The present results are in accordance with these studies regarding *e*.*g*. the higher apparent *in vivo* elongase, Δ5 and Δ6 desaturase activities for fish fed LO.

The present results indicated reduced *fads2* expression in liver and intestine with the temperature increase. Moreover, *elovl5* expression was also reduced in liver of fish fed FO. These results are consistent with those of Norambuena *et al*. [33] on Atlantic salmon held at 10°C or 20°C, which showed reduced hepatic elongase (*elovl2*) and *fads2* gene expressions with increased temperature. In contrast, similar ∆6 desaturase mRNA levels were measured at 16°C and 22°C in European sea bass larvae fed a n-3 LC-PUFA deprived diet [39]. In contrast to the impact of temperature, the diet had no effect on f*ads2* and e*lovl5* expressions. These results are in contradiction with previous studies on salmonids that reported increased desaturase and elongase gene expressions in fish fed plant-derived oils due to the reduced dietary levels of n-3 LC-PUFA, that suppress the expression of these genes [31, 53, 54, 60, 61]. Moreover, several studies already reported that the replacement of fish oil by plant-derived oils increased Δ6 desaturase gene mRNA levels in freshwater fish (see review [53]).

Consistent with the results obtained on the effect of temperature on f*ads2* expression, microsomal Δ6 desaturase activity measured in the intestine of fish fed LO was reduced with the temperature increase. An increase in water temperature had been previously reported to reduce desaturase activity in freshwater fish [35, 62, 63]. This effect was in line with the importance of that enzyme for increased cell membrane fluidity associated with cold acclimation. In accordance, Hagar and Hazel [62] observed increased Δ6 desaturase activity in liver microsomes of rainbow trout when acclimating from 20°C to 5°C. De Torrengo and Brenner [63] also reported that Δ6 desaturase activity was increased in liver microsomes of catfish (*Pimelodus maculatus*) in case of cold acclimation. Furthermore, in rainbow trout, a drop in the desaturation enzymatic activity in hepatocytes and enterocytes has been observed in fish raised at 15°C as compared to 7°C and 11°C [35]. In contrast with the temperature impact, no effect of dietary treatment on microsomal Δ6 desaturase enzymatic activity was observed. In accordance with the present study, no difference in Δ6 desaturase activity was observed in liver and intestinal microsomes of Eurasian perch (*Perca fluviatilis*) fed fish oil or linseed oil diets [49]. However, our results are conflicting with most of the previous studies reporting that Δ6 desaturase activity is under nutritional regulation in fish. Indeed, increased Δ6 desaturase activity was observed in liver microsomes of rainbow trout fed an olive oil diet low in n-3 LC-PUFA, compared to fish fed a fish oil diet [52]. Moreover, numerous studies on isolated hepatocytes showed that plant-derived oil diets consistently induced higher Δ6 desaturase activity in freshwater fish (see review [53]). The discrepancy between these results could be explained by the different methods used (microsome assay *vs* isolated hepatocyte assay). Our results lead us to conclude that, when measured in microsomes, dietary influences on Δ6 desaturase activity were not apparent. It could be interesting to repeat the analysis on isolated hepatocytes in order to study also the impact of cellular environment on Δ6 desaturase activity.

Overall, the present results indicate a negative impact of a temperature increase close to the upper limit of the species temperature tolerance range on the feed efficiency and the fatty acid metabolism of rainbow trout fed a linseed oil diet. Previous studies had reported that rainbow trout possesses a good fatty acid bioconversion capacity at its optimal growth temperature [29, 30, 53] and that this capacity was increased in case of cold acclimation, altering cell membrane fluidity [62, 64]. In warm acclimation, the increased fluidity is unnecessary and the bioconversion capacity not stimulated. Rather than no detrimental effect, increased temperature induced a pronounced negative impact on fish metabolism with decreased desaturase and elongase gene expression and reduced Δ6 desaturase enzymatic activity. To a lesser extent, a negative effect of temperature was also indicated by slightly reduced apparent *in vivo* enzymatic activity of Δ5 and Δ6 desaturases. This less obvious effect emphasised the basal strong capacity of rainbow trout to endogenously produce n-3 LC-PUFA from the n-3 precursor ALA and a particular resistance of this species to external influences, such as water temperature. Despite the detrimental temperature effects on fatty acid metabolism, the whole fish n-3 LC-PUFA content was only slightly affected by temperature. However, a longer trial under similar experimental conditions with fish reaching a marketable size should nevertheless be performed to support the present results and lead to a final conclusion on the water temperature impact, in a human nutrition perspective. In contrast to the temperature effect, dietary lipid source replacement greatly reduced the n-3 LC-PUFA content of rainbow trout. Despite the positive response of fish to the ALA-rich linseed oil diet in terms of increased apparent *in vivo* desaturase and elongase activities, the EPA and DHA contents in fish fed LO did not match those in fish fed FO. More research is thus required on the replacement of fish oil by readily available, economically and environmentally sustainable lipid source alternatives in aquaculture feeds. In this context, studies on transgenic plants rich in EPA and DHA [65], finishing diets [18, 23, 24] or modulators [66, 67] added to feed to improve fish lipid bioconversion capacity could be potential options.

## Supporting Information

S1 Table Apparent digestibility coefficients (%) of fatty acids for the control diet (FO) and the linseed oil diet (LO) used to determine appearance and disappearance of fatty acids (Table 7) and apparent *in vivo* fatty acid metabolism (Table 8). (XLSX)
